# *N*-Benzyl-3-nitro­aniline

**DOI:** 10.1107/S1600536811042632

**Published:** 2011-10-22

**Authors:** Vladimir Stilinović, Tomislav Portada

**Affiliations:** a Laboratory of General and Inorganic Chemistry, Department of Chemistry, Faculty of Science, University of Zagreb, Horvatovac 102 A, HR-10000 Zagreb, Croatia; b Department of Organic Chemistry and Biochemistry, Ruder Bošković Institute, PO Box 180, HR-10002 Zagreb, Croatia

## Abstract

The mol­ecule of the title compound, C_13_H_12_N_2_O_2_, has a bent conformation with a torsion angle about the central C—N bond of 72.55 (19)°. In the crystal, the mol­ecules are connected *via* classical N—H⋯O and non-classical C—H⋯O hydrogen bonds into chains along [10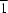]. The dihedral angle between the ring planes is 86.0 (6)°.

## Related literature

For the synthesis of the title compound, see: Magyarfalvi (2008 ▶). For related structures, see: Betz *et al.* (2011 ▶); Iwasaki *et al.* (1988 ▶).
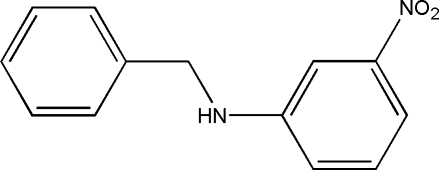


## Experimental

### 

#### Crystal data

C_13_H_12_N_2_O_2_
*M* *_r_* = 228.25
Monoclinic, 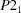

*a* = 5.3359 (2) Å
*b* = 19.2285 (6) Å
*c* = 5.6017 (2) Å
β = 97.334 (3)°
*V* = 570.04 (3) Å^3^
*Z* = 2
Mo *K*α radiation
μ = 0.09 mm^−1^
*T* = 295 K
0.41 × 0.29 × 0.23 mm

#### Data collection

Oxford Diffraction Xcalibur CCD diffractometer
7072 measured reflections
1280 independent reflections
1093 reflections with *I* > 2σ(*I*)
*R*_int_ = 0.017

#### Refinement

*R*[*F* ^2^ > 2σ(*F* ^2^)] = 0.034
*wR*(*F* ^2^) = 0.081
*S* = 1.01
1280 reflections
158 parameters
3 restraints
H atoms treated by a mixture of independent and constrained refinement
Δρ_max_ = 0.14 e Å^−3^
Δρ_min_ = −0.26 e Å^−3^

### 

Data collection: *CrysAlis CCD* (Oxford Diffraction, 2008 ▶); cell refinement: *CrysAlis RED* (Oxford Diffraction, 2008 ▶); data reduction: *CrysAlis RED*; program used to solve structure: *SHELXS97* (Sheldrick, 2008 ▶); program used to refine structure: *SHELXL97* (Sheldrick, 2008 ▶); molecular graphics: *ORTEP-3* (Farrugia, 1997 ▶), *POV-RAY* (Cason *et al.*, 2002 ▶) and *Mercury* (Macrae *et al.*, 2006 ▶); software used to prepare material for publication: *WinGX* (Farrugia, 1999 ▶), *PLATON* (Spek, 2009 ▶) and *PARST* (Nardelli, 1995 ▶).

## Supplementary Material

Crystal structure: contains datablock(s) global, I. DOI: 10.1107/S1600536811042632/rk2302sup1.cif

Structure factors: contains datablock(s) I. DOI: 10.1107/S1600536811042632/rk2302Isup2.hkl

Supplementary material file. DOI: 10.1107/S1600536811042632/rk2302Isup3.cml

Additional supplementary materials:  crystallographic information; 3D view; checkCIF report

## Figures and Tables

**Table 1:** Hydrogen-bond geometry (Å, °)

*D*—H⋯*A*	*D*—H	H⋯*A*	*D*⋯*A*	*D*—H⋯*A*
N1—H1⋯O1^i^	0.84 (2)	2.44 (2)	3.263 (2)	166 (2)
C6—H6⋯O2^i^	0.93	2.45	3.364 (2)	169
C13—H13⋯O1^ii^	0.93	2.67	3.293 (2)	125
C7—H7*A*⋯O1^iii^	0.97	2.61	3.318 (2)	130

Symmetry codes: (i) 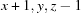; (ii) 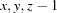; (iii) 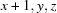.

**Fig. 1.**
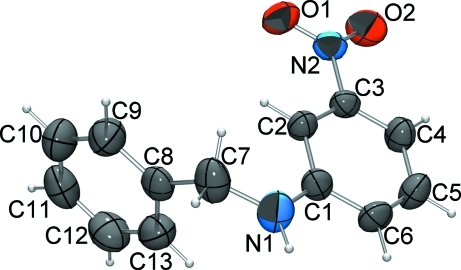
The molecular structure of title compound with the atom labeling scheme. Displacement ellipsoids are shown at 30% probability level. Hydrogen atoms are shown as a spheres of arbitrary radius.

**Fig. 2.**
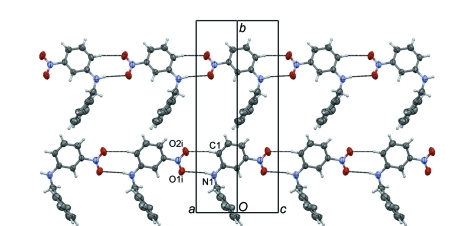
Chains in the structure of the title compound viewed along the [1 0 1] direction. Hydrogen bonds are indicated by dashed lines.

## supplementary crystallographic information

### Comment

The synthesis of the title compound was a part of Preparatory problems for the 40th International Chemistry Olympiad (Magyarfalvi, 2008). It was prepared and crystalized as a part of the laboratory work with high–school students.

The *N*–methyl–3–nitroaniline system is almost perfectly planar (the mean plane of the the phenyl ring is at an angle of 1.5 (2)° with the mean plane of the angle nitro group, and 1.4 (2)° with the mean plane of the central C1—N1—C7. The phenyl substituent is at an angle of 86.0 (2)° to the rest of the molecule. The C1—N1—C7—C8 torsion angle is 72.55 (19)°. This conforamtion is similar to thet reported by Betz *et al.* (2011) for the unsubstituited *N*–benzylaniline.

The molecules are connected into chains along the [1 0 1] direction by N1—H1···O1^i^ classical hydrogen bonds of (N1···O1^i^ = 3.263 (2)Å), which are further fortified by non–classical one C6—H6···O2^i^ with contact C6···O2^ii^ of 3.364 (2)Å. Interestingly, in a similar compound, *N*–benzyl–4–nitroaniline (Iwasaki *et al.*, 1988), the N—H···O bonding is entirely absent, which demonstrates the effect of changing the position of the nitro group on tha supramolecular aggregation.

The chains are further interconnected *via* long non–classical C—H···O (C13—H13···O1^ii^ of 3.293 (2)Å and C7—H7A···O1^iii^ of 3.318 (1)Å) contacts into layers perpendicular to the *b* axis.

Symmetry codes: (i) *x*+1, *y*, *z*-1; (ii) *x*, *y*, *z*-1; (iii) *x*+1, *y*, *z*.

### Experimental

The title compound was prepared using a slightly modified procedure (Magyarfalvi, 2008) and isolated in a form of yellow crystalline powder. A sample of approximately 100 mg of the compound was dissolved in approximately 10 ml of hot ethanol. The solution was left to cool to room temperature, filtered, and the filtrate was left to crystallize by slow evaporation. The single crystalls suitable for X–ray study were obtained after aproximatelly 4 days.

### Refinement

The amine hydrogen atom was locates from the electron fifference map and isotropical refined. All H atoms bonded to carbon atoms were placed geometrically and included in the refinement in the riding–model approximation with C—H distances of 0.93Å for aryl and 0.97Å for CH_2_. In the refinement these H atoms were included with *U*_iso_ = 1.2*U*_eq_(C).

Since there are no heavy atoms in the structure, the Friedel pairs (1186) were merged for the final refinement.

### Crystal data

**Table:**

C_13_H_12_N_2_O_2_	*Z* = 2
*M**_r_* = 228.25	*F*(000) = 240
Monoclinic, *P*2_1_	*D*_x_ = 1.33 Mg m^−^^3^
Hall symbol: P 2yb	Mo *K*α radiation, λ = 0.71073 Å
*a* = 5.3359 (2) Å	θ = 4.6–54.0°
*b* = 19.2285 (6) Å	µ = 0.09 mm^−^^1^
*c* = 5.6017 (2) Å	*T* = 295 K
β = 97.334 (3)°	Plate, yellow
*V* = 570.04 (3) Å^3^	0.41 × 0.29 × 0.23 mm

### Data collection

**Table:**

Oxford Diffraction Xcalibur CCD diffractometer	1093 reflections with *I* > 2σ(*I*)
Radiation source: fine–focus sealed tube	*R*_int_ = 0.017
graphite	θ_max_ = 27.0°, θ_min_ = 3.8°
φ and ω scans	*h* = −6→6
7072 measured reflections	*k* = −24→24
1280 independent reflections	*l* = −7→7

### Refinement

**Table:**

Refinement on *F*^2^	Primary atom site location: structure-invariant direct methods
Least-squares matrix: full	Secondary atom site location: difference Fourier map
*R*[*F*^2^ > 2σ(*F*^2^)] = 0.034	Hydrogen site location: inferred from neighbouring sites
*wR*(*F*^2^) = 0.081	H atoms treated by a mixture of independent and constrained refinement
*S* = 1.01	*w* = 1/[σ^2^(*F*_o_^2^) + (0.0615*P*)^2^] where *P* = (*F*_o_^2^ + 2*F*_c_^2^)/3
1280 reflections	(Δ/σ)_max_ = 0.003
158 parameters	Δρ_max_ = 0.14 e Å^−^^3^
3 restraints	Δρ_min_ = −0.26 e Å^−^^3^

### Special details

**Table:**

Geometry. All s.u.'s (except the s.u. in the dihedral angle between two l.s. planes) are estimated using the full covariance matrix. The cell s.u.'s are taken into account individually in the estimation of s.u.'s in distances, angles and torsion angles; correlations between s.u.'s in cell parameters are only used when they are defined by crystal symmetry. An approximate (isotropic) treatment of cell s.u.'s is used for estimating s.u.'s involving l.s. planes.
Refinement. Refinement of *F*^2^ against ALL reflections. The weighted *R*–factor *wR* and goodness of fit *S* are based on *F*^2^, conventional *R*–factors *R* are based on *F*, with *F* set to zero for negative *F*^2^. The threshold expression of *F*^2^ > σ(*F*^2^) is used only for calculating *R*–factors(gt) *etc*. and is not relevant to the choice of reflections for refinement. *R*–factors based on *F*^2^ are statistically about twice as large as those based on *F*, and *R*–factors based on ALL data will be even larger.

### Fractional atomic coordinates and isotropic or equivalent isotropic displacement parameters (Å^2^)

**Table:**

	*x*	*y*	*z*	*U*_iso_*/*U*_eq_	
H1	0.844 (5)	0.2074 (13)	0.076 (5)	0.063 (6)*	
C3	0.2527 (3)	0.28407 (9)	0.4595 (3)	0.0412 (4)	
C2	0.4146 (3)	0.23191 (9)	0.4090 (3)	0.0423 (4)	
H2	0.4181	0.1896	0.4896	0.051*	
C1	0.5737 (3)	0.24414 (9)	0.2335 (3)	0.0454 (4)	
N2	0.0876 (3)	0.27154 (8)	0.6441 (3)	0.0489 (4)	
C6	0.5584 (3)	0.30889 (10)	0.1187 (4)	0.0516 (4)	
H6	0.6626	0.318	0.0013	0.062*	
C4	0.2364 (4)	0.34794 (9)	0.3472 (4)	0.0528 (5)	
H4	0.1244	0.382	0.3858	0.063*	
N1	0.7446 (4)	0.19560 (9)	0.1759 (4)	0.0663 (5)	
O2	−0.0522 (3)	0.31801 (9)	0.6928 (3)	0.0763 (5)	
O1	0.0942 (3)	0.21462 (8)	0.7433 (3)	0.0645 (4)	
C5	0.3954 (4)	0.35886 (10)	0.1740 (4)	0.0579 (5)	
H5	0.3905	0.4013	0.0939	0.069*	
C8	0.5655 (3)	0.07735 (9)	0.2164 (3)	0.0496 (4)	
C7	0.7768 (4)	0.12849 (11)	0.2881 (5)	0.0616 (5)	
H7A	0.7937	0.1347	0.4612	0.074*	
H7B	0.9335	0.1085	0.2492	0.074*	
C11	0.1852 (4)	−0.01992 (12)	0.0930 (5)	0.0705 (6)	
H11	0.0576	−0.0523	0.0518	0.085*	
C13	0.4114 (4)	0.08153 (12)	−0.0021 (3)	0.0666 (6)	
H13	0.4348	0.1172	−0.1093	0.08*	
C10	0.3354 (5)	−0.02457 (12)	0.3060 (5)	0.0739 (6)	
H10	0.3118	−0.0606	0.4115	0.089*	
C9	0.5222 (4)	0.02354 (12)	0.3673 (4)	0.0651 (5)	
H9	0.6224	0.0197	0.5152	0.078*	
C12	0.2233 (5)	0.03297 (16)	−0.0607 (4)	0.0764 (6)	
H12	0.1207	0.0363	−0.2075	0.092*	

### Atomic displacement parameters (Å^2^)

**Table:**

	*U*^11^	*U*^22^	*U*^33^	*U*^12^	*U*^13^	*U*^23^
C3	0.0386 (7)	0.0460 (9)	0.0405 (8)	−0.0039 (7)	0.0116 (6)	−0.0028 (6)
C2	0.0431 (8)	0.0405 (8)	0.0456 (9)	−0.0033 (7)	0.0142 (7)	0.0021 (7)
C1	0.0417 (8)	0.0458 (9)	0.0515 (10)	−0.0066 (7)	0.0164 (7)	−0.0044 (7)
N2	0.0472 (8)	0.0573 (9)	0.0444 (8)	−0.0030 (7)	0.0150 (6)	−0.0023 (7)
C6	0.0508 (10)	0.0569 (10)	0.0510 (10)	−0.0126 (9)	0.0211 (8)	0.0032 (8)
C4	0.0524 (10)	0.0441 (10)	0.0643 (12)	0.0042 (8)	0.0163 (9)	0.0017 (8)
N1	0.0602 (10)	0.0540 (10)	0.0943 (13)	0.0002 (8)	0.0470 (10)	−0.0012 (9)
O2	0.0773 (10)	0.0795 (11)	0.0811 (11)	0.0173 (9)	0.0447 (9)	−0.0031 (8)
O1	0.0718 (9)	0.0671 (9)	0.0601 (8)	−0.0025 (7)	0.0301 (7)	0.0108 (7)
C5	0.0603 (11)	0.0484 (11)	0.0671 (12)	−0.0028 (9)	0.0164 (10)	0.0147 (8)
C8	0.0481 (9)	0.0457 (10)	0.0577 (10)	0.0089 (8)	0.0171 (8)	−0.0069 (7)
C7	0.0468 (10)	0.0517 (10)	0.0892 (16)	0.0051 (8)	0.0201 (10)	−0.0067 (10)
C11	0.0620 (12)	0.0589 (12)	0.0913 (17)	−0.0032 (10)	0.0128 (12)	−0.0219 (9)
C13	0.0778 (14)	0.0645 (12)	0.0581 (13)	0.0059 (11)	0.0112 (11)	0.0051 (9)
C10	0.0851 (16)	0.0572 (12)	0.0803 (16)	−0.0117 (12)	0.0134 (13)	0.0030 (11)
C9	0.0722 (12)	0.0600 (12)	0.0617 (12)	−0.0029 (11)	0.0030 (10)	0.0041 (10)
C12	0.0778 (14)	0.0816 (14)	0.0653 (13)	0.0054 (13)	−0.0079 (11)	−0.0111 (10)

### Geometric parameters (Å, °)

**Table:**

C3—C2	1.376 (2)	C5—H5	0.93
C3—C4	1.378 (2)	C8—C9	1.374 (3)
C3—N2	1.461 (2)	C8—C13	1.3868 (17)
C2—C1	1.398 (2)	C8—C7	1.511 (3)
C2—H2	0.93	C7—H7A	0.97
C1—N1	1.372 (2)	C7—H7B	0.97
C1—C6	1.399 (3)	C11—C10	1.353 (4)
N2—O2	1.217 (2)	C11—C12	1.364 (4)
N2—O1	1.226 (2)	C11—H11	0.93
C6—C5	1.358 (3)	C13—C12	1.379 (4)
C6—H6	0.93	C13—H13	0.93
C4—C5	1.384 (3)	C10—C9	1.371 (3)
C4—H4	0.93	C10—H10	0.93
N1—C7	1.436 (3)	C9—H9	0.93
N1—H1	0.85 (3)	C12—H12	0.93
			
C2—C3—C4	124.08 (15)	C9—C8—C13	117.32 (18)
C2—C3—N2	118.44 (14)	C9—C8—C7	120.31 (17)
C4—C3—N2	117.48 (15)	C13—C8—C7	122.36 (19)
C3—C2—C1	118.37 (15)	N1—C7—C8	115.13 (19)
C3—C2—H2	120.8	N1—C7—H7A	108.5
C1—C2—H2	120.8	C8—C7—H7A	108.5
N1—C1—C2	122.14 (17)	N1—C7—H7B	108.5
N1—C1—C6	119.94 (16)	C8—C7—H7B	108.5
C2—C1—C6	117.90 (16)	H7A—C7—H7B	107.5
O2—N2—O1	122.47 (15)	C10—C11—C12	119.1 (2)
O2—N2—C3	118.66 (16)	C10—C11—H11	120.4
O1—N2—C3	118.87 (14)	C12—C11—H11	120.4
C5—C6—C1	121.74 (16)	C12—C13—C8	120.2 (2)
C5—C6—H6	119.1	C12—C13—H13	119.9
C1—C6—H6	119.1	C8—C13—H13	119.9
C3—C4—C5	116.54 (16)	C11—C10—C9	120.4 (2)
C3—C4—H4	121.7	C11—C10—H10	119.8
C5—C4—H4	121.7	C9—C10—H10	119.8
C1—N1—C7	123.70 (18)	C10—C9—C8	121.9 (2)
C1—N1—H1	117.6 (17)	C10—C9—H9	119.1
C7—N1—H1	118.6 (17)	C8—C9—H9	119.1
C6—C5—C4	121.36 (17)	C11—C12—C13	121.1 (2)
C6—C5—H5	119.3	C11—C12—H12	119.4
C4—C5—H5	119.3	C13—C12—H12	119.4

### Hydrogen-bond geometry (Å, °)

**Table:**

*D*—H···*A*	*D*—H	H···*A*	*D*···*A*	*D*—H···*A*
N1—H1···O1^i^	0.84 (2)	2.44 (2)	3.263 (2)	166 (2)
C6—H6···O2^i^	0.93	2.45	3.364 (2)	169
C13—H13···O1^ii^	0.93	2.67	3.293 (2)	125
C7—H7A···O1^iii^	0.97	2.61	3.318 (2)	130

Symmetry codes: (i) *x*+1, *y*, *z*−1; (ii) *x*, *y*, *z*−1; (iii) *x*+1, *y*, *z*.
